# Screening for cancers with a good prognosis: The case of testicular germ cell cancer

**DOI:** 10.1002/cam4.3837

**Published:** 2021-03-12

**Authors:** Eveline A. M. Heijnsdijk, Steven J. Supit, Leendert H. J. Looijenga, Harry J. de Koning

**Affiliations:** ^1^ Department of Public Health Erasmus MC University Medical Center Rotterdam Rotterdam The Netherlands; ^2^ Princess Máxima Center for Pediatric Oncology Utrecht The Netherlands

**Keywords:** epidemiology, risk model, screening, urological oncology

## Abstract

**Background:**

To determine, using testicular germ cell cancer screening as an example, whether screening can also be effective for cancers with a good prognosis.

**Methods:**

Based on the Dutch incidence, stage distribution, and survival and mortality data of testicular germ cell cancer, we developed a microsimulation model. This model simulates screening scenarios varying in screening age, interval, self‐examination or screening by the general practitioner (GP), and screening of a defined high‐risk group (cryptorchidism). For each scenario, the number of clinically and screen‐detected cancers by stage, referrals, testicular germ cell cancer deaths, and life‐years gained were projected.

**Results:**

Annual self‐examination from age 20 to 30 years resulted in 767 cancers detected per 100,000 men followed over life‐time, of which 123 (16%) by screening. In this scenario, 19.2 men died from the disease, 4.7 (20%) less than without screening, and 230 life‐years were gained. Around 14,000 visits to the GP and 2080 visits to an urologist were required. This scenario resulted in the most favorable ratio between extra visits to the GP or urologist and deaths prevented (1418 and 116 respectively). Monthly screening, or screening until age 40 resulted in less favorable ratios. Self‐examination by only the high‐risk population prevented 1.0 death per 100,00 men in the general population. In all scenarios, 46–50 life‐years were gained for each testicular germ cell cancer death prevented.

**Conclusion:**

Despite the good prognosis, self‐examination at young ages for testicular germ cell cancer could be considered.

## Graphical abstract

In this study, we describe possible screening strategies to screen for testicular germ cell cancer. With the use of a model calibrated to the Dutch situation, we simulated several screening strategies. We found that despite the good prognosis, self‐examination at young ages for testicular germ cell cancer could be considered.

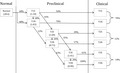

## 1 INTRODUCTION

Screening for breast, cervical, and colorectal cancer has been shown to lead to a reduction in cancer‐specific mortality.^1^, ^2^, ^3^ In many countries organized or opportunistic screening programs are available for these three types of cancer. In general, screening will reduce the number of cases detected in an advanced stage. Therefore, it might be debatable whether screening can also be effective for cancers with an overall good prognosis, for example, testicular germ cell cancer, which is rare and, contrary to most cancer types, generally detected in young adults. Testicular germ cell cancer accounts for approximately only 1% of all male cancers, but in developed countries it is the most common cancer in young adult men aged 15–44 years.^4^ In the United States, the incidence is largest in non‐Hispanic white men (6.97 per 100,000 men‐years) and lowest in black men (1.20 per 100,000 men‐years).^5^ The incidence of testicular germ cell cancer is increasing worldwide over the past two decades, for which the reasons are still largely unknown.^4^, ^6^ Cryptorchidism is an important risk factor for testicular germ cell cancer, confirmed in a meta‐analysis of 21 studies demonstrating a relative risk of developing testicular germ cell cancer of 4.8.^7^

Most men with testicular germ cell cancer present symptomatically with a palpable lump that may or may not be painful.^6^ There is an average delay of 3 to 5 months (range 1–36 months) between initial symptoms and final diagnosis,^8^, ^9^ which probably has an impact on survival. When the cancer is detected in a localized stage (stage I; 68% of the cases detected), the 5‐year survival is 99.0%. For distant disease (12% of cases detected), the 5‐year survival decreases to 72.5%. Overall, testicular germ cell cancer has a good prognosis, showing a 5‐year survival of 95.0%.^10^ There are 20 to 30 testicular germ cell cancer–related deaths each year in the Netherlands and around 1,650 in Europe.^11^

Screening for testicular germ cell cancer can be performed by a general practitioner (GP) or by self‐examination. However, for both screening set ups the sensitivity is unknown.^9^ Potential harms associated with screening include false‐positive results, anxiety, and harms from diagnostic tests or procedures. So far, no randomized trials or observational studies have been performed to quantify the benefits and harms of testicular germ cell cancer screening.

The United States Preventive Service Task Force (USPSTF) does not recommend screening for testicular germ cell cancer in asymptomatic adolescents or adults (grade D) based on the lack of adequate evidence that screening by either clinical examination or patient self‐examination has a higher yield or greater accuracy for detecting testicular germ cell cancer at earlier stages. In addition, there is moderate certainty that screening for this type of cancer has no net benefit.^12^ In contrast, several papers have published against this recommendation, arguing that there is no evidence of any harm, and that false positives may have a significant function in finding other diseases and that anxiety. Also, false positives can be reduced using guidelines and patient education.^13^, ^14^, ^15^

Based on this current status of evidence, this study was performed with the aim to estimate which factors mostly influence the harms and benefits of testicular germ cell cancer screening.

## 2 METHODS

### 2.1 MIcrosimulation SCreening ANalysis (MISCAN) model

**FIGURE 1:**
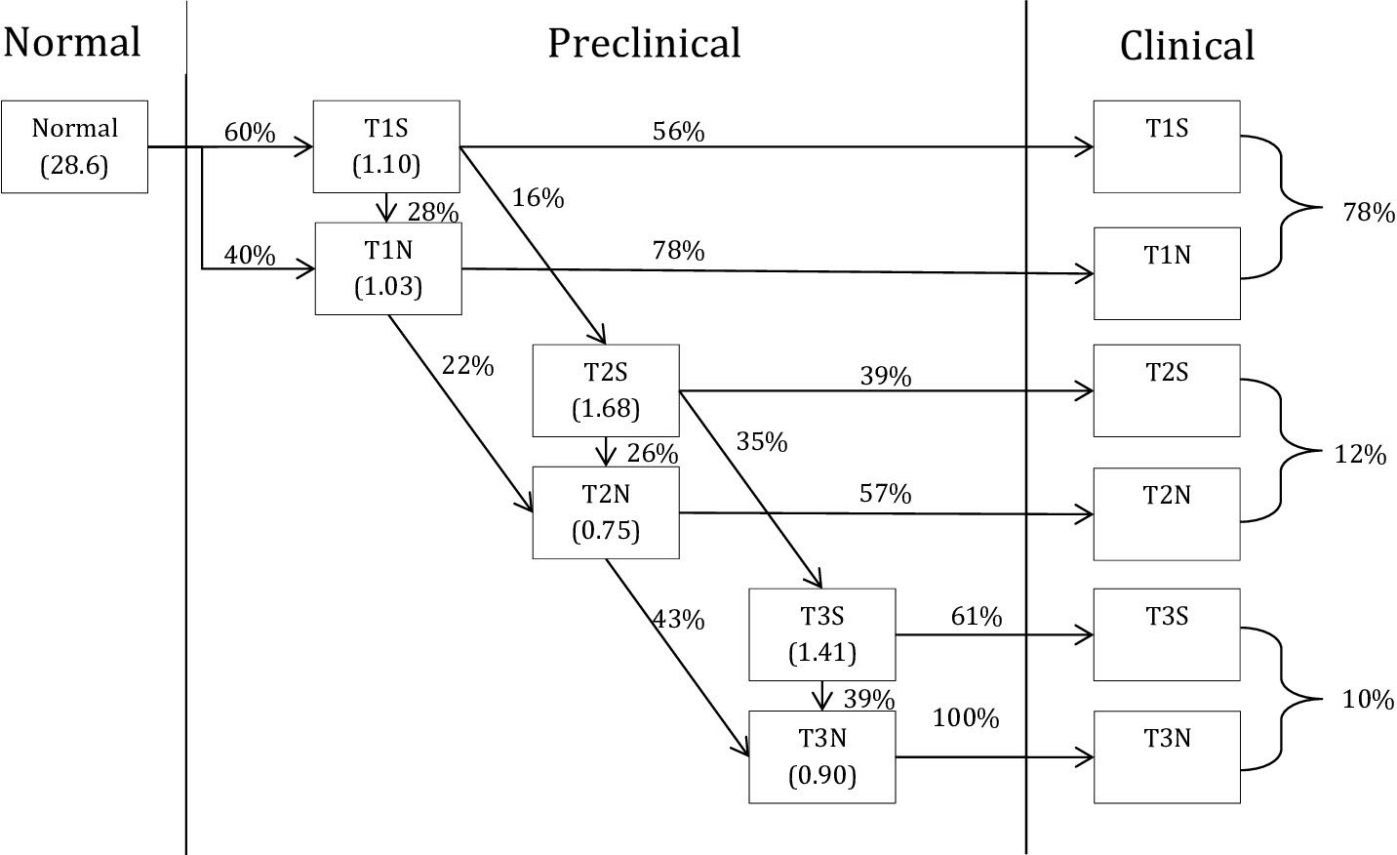
General structure of the MISCAN model including the natural history of testicular germ cell cancer prior to clinical diagnosis. In the preclinical phase, the cancer can also be detected by screening. Preclinical transition probabilities are indicated next to the arrows. Mean dwelling times of the preclinical stage (exponential distributions) are indicated in the boxes in years. The predicted stage distribution in case of no screening is presented on the right

We developed a microsimulation model using the MISCAN framework to simulate the impact of testicular germ cell cancer screening on mortality. MISCAN is a microsimulation model developed for the evaluation of screening and has been used previously to estimate the harms, benefits, and cost‐effectiveness of, for example, breast, colorectal, cervical, and prostate cancer screening.^16^, ^17^, ^18^, ^19^ In the testicular germ cell cancer model, individual life histories of men are simulated by modeling the transitions between possible health stages (Figure 1). All men start in the no testicular cancer stage, although possibly at risk. Some men will develop testicular germ cell cancer, which can be a seminoma or non‐seminoma (possibly also containing a seminoma component). Although rare, a seminoma can progress into a non‐seminoma, and all testicular germ cell cancers can progress from preclinical stage T1 to T2 to T3. In each stage, the cancer can be clinically detected or detected by screening. Based on Dutch data, a 5‐year survival of 99% was assumed for T‐stage 1, 99% for T2, and 89% for T3.^20^ The probability of other causes of death is based on the Dutch male life table for 2016.^21^

The probabilities of onset by age, the durations of the various stages (exponential distributions, to allow for short as well as long durations), and the transition probabilities to progress from one stage to another are estimated on incidence data by 5‐year age group and stage distribution data of the Netherlands in the period 1991–2019^20^ using the Nelder‐Mead calibration method. To account for the increasing trend in incidence over the years, an increasing onset rate over the years 1991–2019 was estimated (1.3% to 4% by year). The projected testicular germ cell cancer mortality was compared with the observed mortality over the period 1996–2017.^20^ The final parameters of the model are presented in Figure 1 and Table 1.

**TABLE 1:** Input parameters of the model

Parameter	Description/Value	Source
Time before clinical detection	Depending on stage, see Figure 1	Calibrated to Dutch incidence and stage distribution data
Transition probability to the next stage	Depending on stage, see Figure 1	Calibrated to Dutch incidence and stage distribution data
Survival	T1 and T2: 99% T3: 89%	Dutch Cancer Registry
Attendance to self‐examination/screening	At each screen, a new random selection of 80% of the eligible population is made	Assumption based on other cancer screening programs
Sensitivity of the screening test	70%	Assumption based on expert opinion
Visits to GP	1% of self‐examinations	Assumption based on expert opinion
Referral to urologist	10% of visits to GP	Assumption based on expert opinion
Diagnostic activities for clinically detected cancers	For each cancer detected clinically, 10 men visited the GP and 2 the urologist	Assumption based on expert opinion
Other cause death	Life table of Dutch men in 2016	Statistics Netherlands

Separately, a population including a cohort of men with cryptorchidism (3% of the population) was simulated by using a 4.8 times higher risk of onset.

### 2.2 Screening scenarios

We used the calibrated MISCAN model to simulate various screening scenarios of self‐examination. Based on existing cancer screening programs we used an 80% compliance at each screen (each screen randomly 80% of the eligible population is participating). The test sensitivity is unknown. We assumed a 70% sensitivity of the self‐examination. We assumed that 1% of the self‐examinations was followed by a visit to the GP and that 10% of the visits to the GP were followed by a referral to an urologist (expert opinion). For each cancer detected clinically, it was assumed that 10 men visited the GP and 2 the urologist (expert opinion). Screening started at age 20 and stopped at age 30 or 40 years. Screening intervals of 1 year, 0.25 year, and 0.08 year (1 month) were evaluated.

Two alternative screening protocols were assessed: annual screening by the GP, with an assumed test sensitivity of 80%, and a protocol in which only the 3% high‐risk population performed self‐examination every 0.25 year.

In addition, three parameters in the model were varied: the sensitivity of the test was varied between 35% and 80%, the 5‐year survival for stage T3 was varied between 80% and 95%, and a 40% attendance to screening was modelled.

All runs were performed using a sample size of 10 million men born in 2000 followed over lifetime. The results were scaled to a cohort of 100,000 men. For each scenario, the number of clinically and screen detected cancers by stage as well as the referrals, the number of testicular germ cell cancer deaths, and the life‐years gained were projected.

## 3 RESULTS

**FIGURE 2:**
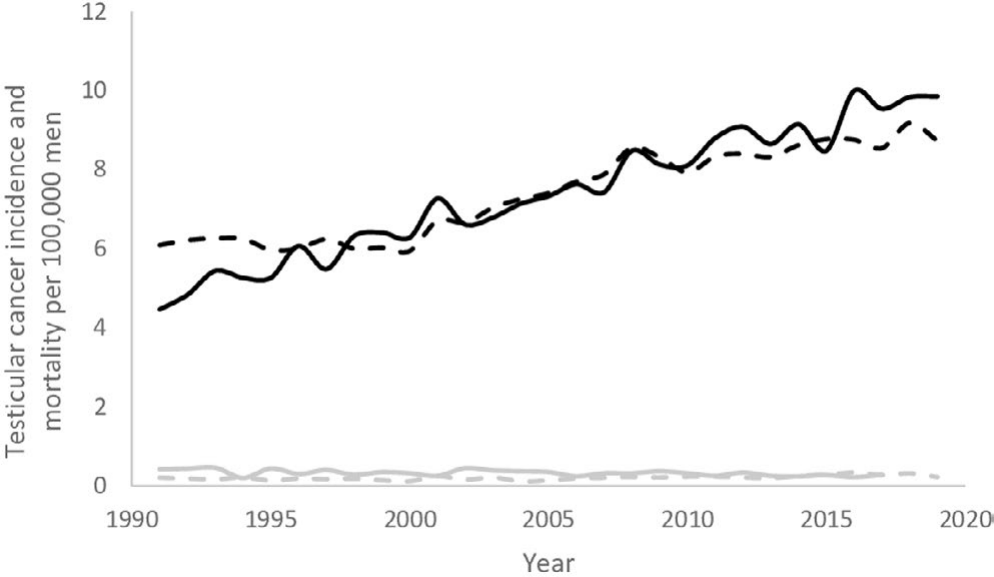
The incidence (black lines) and mortality (grey lines) of testicular germ cell cancer in the Netherlands in the period 1991 to 2019 (solid lines) compared with the model predictions (dashed lines). The numbers are crude rates per 100,000 men

The model projected the testicular germ cell cancer incidence (as well as the trend in incidence) and mortality in the Netherlands well (Figure 2). Projections by 5‐year age group are presented in the Appendix. The predicted stage distribution was 78% in stage T1, 12% in stage T2, and 10% in stage T3, compared with 75%, 14%, and 11% observed, respectively.

In a no‐screen scenario, the calibrated model predicted 766 testicular cancers detected per 100,000 men followed over lifetime, of which 73 were detected in stage T3 (Table 2). In this scenario, 23.9 testicular germ cell cancer deaths were predicted.

**TABLE 2:** Predicted effects of various screening scenarios for a birth cohort of 100,000 men followed over lifetime

Screening age (years)	No	20–30	20–30	20–30	20–40	20–30	20–30
Interval (years)	‐	1	0.25	0.08	0.25	1	0.25
Scenario						Screening by GP	High‐risk only
Self‐examinations	0	792,000	3,165,000	9,454,000	6,306,000	0	94,000
Visits to GP	7664	14,358	37,242	99,755	67,039	797,833	8308
Visits to urologist	1533	2080	4283	10,496	7101	1800	1568
Clinically detected cancers							
T1	600	513	444	411	322	504	578
T2	93	75	65	62	44	73	89
T3	73	56	49	47	31	55	70
Screen detected cancers							
T1	0	111	199	240	356	122	29
T2	0	9	7	4	10	9	1
T3	0	3	2	1	3	3	0
% screen detected	0%	16%	27%	32%	48%	17%	4%
Total number of testicular cancers diagnosed	766	767	767	767	767	767	767
Testicular cancer deaths	23.9	19.2	16.8	15.9	11.7	18.9	22.8
Testicular cancer mortality reduction	0	4.7 (20%)	7.1 (30%)	8.0 (34%)	12.2 (51%)	5.0 (21%)	1.1 (4%)
Life‐years gained	0	230	350	400	580	250	50
Additional visits GP/TC deaths prevented	0	1418	4160	11,397	4867	158,044	636
Additional visits urologist/TC deaths prevented	0	116	387	1109	456	105	34

Annual self‐examination from the age of 20 to 30 years resulted in 767 testicular cancers detected, of which 123 (16%) by screening. In this scenario, 59 testicular germ cell cancers were detected in stage T3 and 19.2 men died from the disease. The testicular germ cell cancer mortality reduction due to screening was 4.7 per 100,000 men (20% reduction) and 230 life‐years were gained. This screening scenario resulted in 14,358 visits to the GP and 2080 visits to an urologist.

In the monthly screening scenario, the testicular germ cell cancer mortality reduction due to screening was 8.1 (34%) and 400 life‐years were gained. Screening until the age of 40 years resulted in a testicular cancer mortality reduction of 12.2 (51%) and 560 life‐years were gained. Both scenarios required substantially more visits to the GP and urologist (Table 2). The self‐examination scenario assuming annual screening between ages 20–30 resulted in the most favorable ratio between extra visits to the GP or urologist and the testicular germ cell cancer deaths prevented (1418 and 116 respectively).

In all scenarios, 46 to 50 life‐years were gained for each testicular germ cell cancer death prevented and the mean lead time (the time screening advances the diagnosis) was around 1.7 years.

Annual screening by the GP reduced the testicular germ cell cancer mortality by 5.0 cases (21%), increased the visits to the GP (158,044 per testicular death prevented), and decreased the visits to the urologist (105 per testicular death prevented). Self‐examination by the high‐risk population only resulted in the most favorable ratio between visits to the urologist and testicular death prevented (34), while only 1.0 testicular germ cell cancer death was prevented in the entire population.

Changing the sensitivity of the test to 35% or 80%, the survival of T3 tumors between 80% and 95% or the attendance to 40% had a smaller impact on the ratio between visits to the urologist and testicular death prevented (Table 3).

**TABLE 3:** Predicted effects of various screening scenarios for a birth cohort of 100,000 men followed over lifetime

Screening age (years)	20–30	20–30	20–30	20–30	20–30
Interval (years)	0.25	0.25	0.25	0.25	0.25
Scenario	35% sensitivity	80% sensitivity	80% survival	95% survival	40% attendance
Self‐examinations	3,165,000	3,164,950	3,165,000	3,165,000	1,583,000
Visits to GP	37,708	37,159	37,242	37,242	21,872
Visits to urologist	4376	4267	4283	4283	2792
Clinically detected cancers					
T1	482	437	444	444	482
T2	70	65	65	65	69
T3	53	49	49	49	53
Screen detected cancers					
T1	150	208	199	199	151
T2	9	6	7	7	9
T3	2	2	2	2	2
% screen detected	21%	28%	27%	27%	21%
Total number of testicular cancers diagnosed	767	767	767	767	767
Testicular cancer deaths^a^	18.2	16.6	21.0	13.5	18.3
Testicular cancer mortality reduction	5.8 (24%)	7.4 (31%)	9.1 (30%)	5.4 (28%)	5.7 (24%)
Life‐years gained	280	360	450	270	280
Additional visits GP/TC deaths prevented	5225	4013	3268	5498	2501
Additional visits urologist/TC deaths prevented	495	372	304	511	222

^a^ Without screening there would be 30.3 testicular germ cell cancer deaths when the survival for T3 would be 80% and 18.9 when the survival for T3 would be 95%.

## 4 DISCUSSION

This study showed that the screening interval, screening ages, and the way screening is organized (self‐examination, by the GP, or only high‐risk groups) had a substantial impact on the testicular germ cell deaths prevented and the number of visits. To the best of our knowledge, this is the first microsimulation model for testicular germ cell cancer screening. The model is based on data from a large nation‐wide population (around 800 cases each year) and shows a good fit, supporting the validity of the microsimulation model as applied. The model will be best generalizable to countries with a predominantly white male population and a relatively high incidence of testicular germ cell cancer, such as countries in Western, Northern, and Southern Europe, North America, and Australia. Due to the lower incidence in non‐white men, screening will be less efficient for other populations.

Limitations of this study are the uncertainty of several parameters in the model, especially sensitivity and specificity of the examination by the person itself, the GP, and the urologist. These parameters are unknown and it has been stated that false‐negative and false‐positive examinations are common even among urologists and would be significantly higher among other practitioners.^9^ In addition, false‐positive findings can lead to the detection of other conditions, of which the harms and benefits are difficult to quantify. However, as indicated, the impact of varying the test sensitivity between 35% and 80% had a smaller impact than the screening interval, protocol, or survival.

Another limitation is that we did not include quality‐adjusted life‐years (QALYs) in this analysis because of lack of data. False positives due to screening will lead to less QALYs, whereas less intensive treatment because advanced disease is averted by screening will lead to a gain in QALYs. The exact value of the utility estimates will determine whether the QALYs gained due to screening will be more or less than the life‐years gained.

We also did not include costs. A previous study calculated that the total treatment costs for an advanced stage testicular germ cell cancer were equal to the costs of 313–330 benign office visits as a result of self‐examination or to the costs of 180–190 office visits with ultrasound.^13^ These numbers are in our study for the annual self‐examination from age 20 to 30 years scenario, 487 additional visits to the GP, and 39 visits to the urologist per stage T3 cancer prevented. No complete cost‐effectiveness studies for screening for testicular germ cell cancer have been performed previously.

Generally, in cancer screening, overdiagnosis and false positives are the most important harms. In the case of testicular germ cell cancer screening, overdiagnosis does not seem to be an important harm, mainly because of the young age at detection and the relatively fast tumor growth. There was only one additional case of testicular germ cell cancer detected per 100,000 men in all screening scenarios modelled, compared to the no screening scenario of 766 cancers detected. False positives are important due to possible anxiety of a visit to the GP or a referral to the urologist. Also, these additional visits will increase healthcare costs.

Compared with the self‐examination scenarios, the alternative scenarios of screening by the GP or self‐examination of the high‐risk group only (i.e., cryptorchidism) resulted indeed in much less visits to the urologist. However, the result also demonstrates that these strategies have important drawbacks as well: the strategy of screening by the GP requires much more visits to the GP, which can probably only be cost‐effective when this visit is combined with another purpose. During such a visit, risk factors or symptoms can be discussed (case finding). The scenario of screening the high‐risk‐only group results in a very small testicular germ cell cancer mortality reduction because this group is only 3% of the total population. Combinations of intensively screening the high‐risk population and less intensively screening the remaining population are also possible.

Apart from cryptorchidism, other risk factors are family history, infertility, and a history of testicular germ cell cancer.^6^ Especially family history, with a 4‐ to 10‐fold higher risk of developing testicular germ cell cancer, can probably be used to stratify screening. However, <10% of the men with testicular germ cell cancer has a family history of the disease. Several single nucleotide polymorphisms (SNPs) have been identified, but these remain of limited use for the general population in the context of screening.^6^

Screening for testicular germ cell cancer can be compared with screening for other cancers, for example, biennial breast cancer screening from the age of 50 to 74 years, or screening for HPV‐based cervical cancer every 5 years from age 30 to 60 years.^18^, ^22^ Although the relative cancer mortality reduction is around 30%, the absolute testicular germ cell cancer mortality is low, with <10 deaths prevented per 100,000 men (1200 breast cancer deaths prevented and 234 cervical cancer deaths prevented per 100,000 women). However, due to the generally young age at detection, the average 50 life‐years gained per death prevented for testicular germ cell cancers are high compared with 13 for breast cancer and 22 for cervical cancer.

In conclusion, due to the good prognosis of testicular germ cell cancer, the absolute mortality reduction by screening is small, with <10 testicular deaths prevented per 100,000 men. Still, about 20% to 50% of the testicular germ cell cancer deaths can be prevented by screening. The screening interval and the organization of the screening (by self‐examination or the GP, or screening of the high‐risk group only) impact the results strongly. However, before testicular germ cell cancer screening can be recommended, more research on the test sensitivity and the number of men referred to the urologist should be conducted, since these model parameters are unknown and were based on assumptions.

## CONFLICTS OF INTEREST

The authors have nothing to disclose.

## AUTHOR CONTRIBUTIONS

EH: model development, analysis, and writing – original draft; SS: data collection, model development, analysis, and writing – review and editing; LL: conceptualization, supervision, and writing – review and editing; HdeK: conceptualization, supervision, and writing – review and editing.

## ETHICAL APPROVAL

No ethical approval was sought for this study since only aggregated, publicly available data were used.

## Supporting information

Supplementary Material

## Data Availability

The data that support the findings of this study are available from the corresponding author upon reasonable request.
