# Supplementary material for: Screening for cancers with a good prognosis: The case of testicular germ cell cancer
Source: Cancer Med. 2021 Mar 12;10(8):2897–903. doi: 10.1002/cam4.3837 (PMC8026933; doi:10.1002/cam4.3837)
Supplement: Supplementary file 1 — Supplementary Material [file CAM4-10-2897-s001.docx]

**Appendix Screening for cancers with a good prognosis: the case of testicular germ cell cancer**

The following graphs present the testicular cancer incidence in the Netherlands (solid lines) and the predicted incidence by the model (dashed lines) by 5-year age groups.
